# Cytokines in patients with Posner-Schlossman syndrome

**DOI:** 10.3389/fimmu.2026.1739027

**Published:** 2026-01-29

**Authors:** Yu Liu, Shengjie Li, Xiangmei Kong, Qilian Sheng, Zhujian Wang, Wenjun Cao

**Affiliations:** 1 Department of Laboratory Medicine, Fudan University Eye Ear Nose and Throat Hospital, Shanghai, China; 2 Department of Ophthalmology, Fudan University Eye Ear Nose and Throat Hospital, Shanghai, China

**Keywords:** aqueous humor, corneal endothelial cell density, cytokines, intraocular pressure, Posner-Schlossman syndrome

## Abstract

**Purpose:**

To investigate the inflammatory cytokine profiles in the aqueous humor (AH) of patients with Posner-Schlossman syndrome (PSS) and evaluate their correlations with key ophthalmic parameters.

**Methods:**

Aqueous humor samples were collected from 31 eyes with PSS, 26 eyes with primary open-angle glaucoma (POAG), and 20 eyes with age-related cataract (ARC, control group) at the Eye and ENT Hospital of Fudan University. A multiplex bead-based flow cytometric immunoassay was performed to quantify the concentrations of interleukins (IL-1β, IL-2, IL-4, IL-5, IL-6, IL-8, IL-10, IL-12, IL-17), tumor necrosis factor-alpha (TNF-α), and interferons (IFN-α, IFN-γ). Simultaneously, the presence of cytomegalovirus (CMV) DNA in PSS samples was assessed by PCR. Clinical data including corneal endothelial cell density, visual acuity, intraocular pressure (IOP), and visual field were also recorded.

**Results:**

The AH levels of IL-1β, IL-5, IL-6, IL-8, IL-10, IFN-γ, and TNF-α were significantly elevated in PSS patients compared to controls (*P* < 0.05), with IL-1β, IL-6, IL-10, and IFN-γ levels also significantly higher than in POAG patients. ROC curve analysis demonstrated diagnostic value of these four cytokines in differentiating PSS from POAG (P<0.05). No significant differences in cytokine levels were observed between CMV DNA-positive and -negative PSS samples. Notably, IL-6 levels positively correlated with IL-8 and IL-10, and also showed significant associations with IOP (r = 0.395, P = 0.007) and relative endothelial cell loss (RECL) (r = 0.453, P = 0.039).

**Conclusion:**

Distinct inflammatory cytokine profiles in the AH of PSS patients suggest a prominent immune response potentially contributing to disease pathogenesis. IL-6 may serve as a biomarker reflecting both inflammation and tissue damage in PSS. Note: CECD, corneal endothelial cell density; RECL, relative decrease in CECD loss between the affected eye and the fellow eye.

## 1 Introduction

Posner-Schlossman syndrome (PSS), first described by Posner and Schlossman in 1948 (1), is a distinct subtype of secondary glaucoma characterized by recurrent episodes of unilateral elevated intraocular pressure (IOP) accompanied by mild anterior uveitis. The fellow eye typically exhibits normal IOP and no signs of inflammation. Clinically, PSS presents with episodic blurred vision, ocular discomfort or pain, and keratic precipitates (KPs) on slit-lamp examination. The syndrome predominantly affects males between 20 and 50 years of age (2). Though bilateral involvement—either simultaneous or alternating—has also been reported in some cases (3, 4).

PSS shares certain clinical features with primary open-angle glaucoma (POAG), particularly in the early stages, which can make differential diagnosis challenging. Atypical presentations or coexisting PSS and POAG further complicate diagnosis. The absence of disease-specific ocular lesions and validated biomarkers often leads to delayed diagnosis, hindering timely and targeted treatment. As the chronic, relapsing nature of PSS becomes increasingly recognized, its associated socioeconomic burden has prompted growing interest in identifying disease-specific biomarkers to support accurate diagnosis and personalized management strategies.

Despite decades of study, the pathogenesis of PSS remains incompletely elucidated. Prior research has suggested that persistent inflammatory responses within the anterior chamber may impair aqueous humor outflow, resulting in elevated IOP and secondary glaucomatous damage. Cytomegalovirus (CMV) infection has been frequently implicated as a contributing factor in this process (5–8). Simultaneously, multiple cytokines have been reported to participate in immune modulation and inflammatory signaling in glaucomatous diseases (9, 10). However, due to the complexity and redundancy of cytokine networks, their precise roles in PSS remain unclear. Therefore, a comprehensive profiling of inflammatory cytokines and their associations with clinical parameters is critical to understanding PSS pathogenesis and identifying potential diagnostic biomarkers.

In this study, we employed multiplex microsphere flow cytometric immunoassay to measure the levels of a panel of inflammatory cytokines in the aqueous humor of PSS patients and compared these profiles to those of POAG and cataract (control) patients. We further explored correlations between cytokine concentrations and ophthalmic indicators—including visual acuity, IOP, and corneal endothelial cell density—to evaluate their diagnostic potential and relevance to disease severity. These findings may lay the groundwork for the development of laboratory-based diagnostic tools and provide mechanistic insights into the immunopathogenesis of PSS.

## 2 Materials and methods

### 2.1 Study subjects

A total of 77 eyes from 77 patients were included in this study, comprising 31 eyes diagnosed with Posner-Schlossman syndrome (PSS), 26 with primary open-angle glaucoma (POAG), and 20 with age-related cataract (ARC, serving as controls). All participants were recruited from the Eye and ENT Hospital, Fudan University, between August 2022 and November 2024. Patients in the control group exhibited no signs of ocular pathology upon comprehensive ophthalmic examination and had intraocular pressure (IOP) values below 21 mmHg.

All participants enrolled in this study were carefully screened and confirmed to have no underlying systemic diseases (e.g., diabetes, autoimmune disorders, cardiovascular diseases, etc.). Furthermore, all samples were collected prior to the initiation of any study-related or relevant medications, ensuring that the sample status reflected the natural history of the condition under investigation without pharmacological confounding. In patients with PSS, aqueous humor sampling was performed during an active episode characterized by elevated intraocular pressure and mild anterior chamber inflammation, but before the initiation of treatment, to ensure that cytokine profiles reflected the intrinsic inflammatory status of the disease.

The diagnosis of PSS was based on the following criteria: (1) recurrent unilateral episodes of elevated IOP (>21 mmHg) with spontaneous return to normal between episodes; (2) mild anterior chamber inflammation, characterized by fine keratic precipitates on slit-lamp examination; (3) open iridocorneal angle confirmed by gonioscopy; and (4) a healthy fellow eye with normal IOP, visual field, and optic disc morphology (11). POAG diagnosis was established according to standard criteria, including an open iridocorneal angle, documented history of elevated IOP prior to anti-glaucoma treatment, and exclusion of secondary glaucoma.

The study protocol adhered to the tenets of the Declaration of Helsinki and was approved by the Institutional Ethics Committee of the Eye and ENT Hospital, Fudan University. Written informed consent was obtained from all participants.

### 2.2 Reagents and instruments

A 12-plex human cytokine detection kit (Catalog No. 191217) was purchased from Hangzhou R&D Biological Technology Co., Ltd. Inflammatory cytokines in aqueous humor—including IL-1β, IL-2, IL-4, IL-5, IL-6, IL-8, IL-10, IL-12, IL-17, TNF-α, IFN-α, and IFN-γ—were measured using a bead-based multiplex flow cytometry immunoassay. The procedure employed a microsphere-based sandwich immunofluorescence technique, integrated with enzyme-linked immunosorbent assay (ELISA) principles.

Qualitative polymerase chain reaction (PCR) for CMV DNA detection was performed using a TaqMan PCR kit (Shanghai ZJ BioTech Co., Ltd., China) on an ABI 7500 real-time PCR system (Thermo Fisher Scientific, Waltham, MA, USA).

### 2.3 Sample collection

Aqueous humor samples (100 μL each) were obtained via anterior chamber paracentesis prior to any intraocular surgery or pharmacological treatment. All samples were collected under aseptic conditions and immediately stored at –80 °C until analysis.

### 2.4 Collection of ocular clinical parameters

Visual Acuity: Visual acuity was measured using an international logMAR visual acuity chart. Visual Field Examination: Visual fields were measured using an Octopus 900 automated perimeter. Corneal Endothelial Cell Density: Corneal endothelial cell density was measured using a corneal endothelial microscope (NSP-9900II).

### 2.5 Statistical analysis

Data analysis was conducted using IBM SPSS Statistics 21.0. The Shapiro–Wilk test was used to assess normality. For normally distributed variables, results are expressed as mean ± standard deviation (SD), and comparisons between groups were made using independent-sample t-tests. Non-normally distributed variables are presented as median with interquartile range [IQR], and analyzed using the Mann–Whitney U test. Categorical variables were compared using Fisher’s exact test.

Spearman’s rank correlation analysis was performed to evaluate associations between cytokine concentrations and clinical parameters (e.g., IOP, CECD, visual field). Correlation strength was defined as: strong (|r| ≥ 0.8), moderate (|r| = 0.5–0.8), weak (|r| = 0.3–0.5), and negligible (|r| < 0.3). A P-value <0.05 was considered statistically significant. Given the exploratory nature of this study and the biological correlations among cytokines, formal correction for multiple comparisons was not applied. The findings should therefore be interpreted with caution and viewed as hypothesis-generating.

Receiver operating characteristic (ROC) curves were generated using MedCalc 19.6 (MedCalc Software Ltd., Ostend, Belgium) to assess the diagnostic performance of candidate cytokines. For ROC curve analyses, optimal cutoff values were determined using Youden’s index, defined as sensitivity + specificity − 1.

## 3 Results

### 3.1 Demographic and clinical characteristics

**Table 1** summarizes the demographic and preoperative clinical characteristics of the study population. There were no statistically significant differences in age or gender distribution among the PSS, POAG, and control groups (*P* > 0.05). Both the PSS and POAG groups exhibited significantly elevated intraocular pressure (IOP) compared with controls (*P* < 0.0001), while the IOP difference between PSS and POAG groups was not statistically significant (*P* = 0.0534).

**Table 1:** Baseline demographic and clinical data among study groups.

Factors	Groups		
	Control	PSS	POAG
No. of eyes	20	31	26
Age (years)	54.4 ± 9.7	50.1 ± 12.5	55.5 ± 12.8
*P* value *vs*. Control ^a^	–	0.2058	0.7310
*P* value *vs*. PSS ^a^	0.2058	–	0.1122
Male:female	12:8	19:12	19:7
*P* value *vs*. Control ^b^	–	0.9273	0.3535
*P* value *vs*. PSS ^b^	0.9273	–	0.3512
IOP (mmHg)	13.8 ± 1.8	32.9 ± 9.9	28.2 ± 7.3
*P* value *vs*. Control ^a^	–	<0.0001	<0.0001
*P* value *vs*. PSS ^a^	<0.0001	–	0.0534

a *P* values are calculated between each pair of groups using the Mann–Whitney U-test.

b *P* values are calculated between each pair of groups using the Fisher’s exact probability test.

### 3.2 Cytokine profiles in aqueous humor

Among the 12 measured cytokines, IL-2, IL-4, IL-12, and IFN-α were below the lower limit of detection in the majority of samples across all three groups and were therefore excluded from subsequent quantitative analyses.

As shown in **Table 2**, the levels of IL-1β, IL-5, IL-6, IL-8, IL-10, IFN-γ, and TNF-α in the aqueous humor of PSS patients were significantly higher than those in the cataract control group (*P* < 0.05). Furthermore, IL-1β, IL-6, IL-10, and IFN-γ levels were also significantly elevated in PSS compared to POAG eyes (*P* < 0.05), while IL-5 and TNF-α showed no significant differences between the two.

**Table 2:** Aqueous humor cytokine levels (pg/ml) in control, PSS, and POAG groups.

Cytokines	Control n= 20	PSS n =31	POAG n =26	*P* value		
				*Control vs PSS*	*Control vs POAG*	*PSS vs POAG*
IL- 1β	1.75 (0.53,2.46)	8.96 (4.89,13.69)	0.77 (0.53,3.14)	<0.0001*	0.6622	<0.0001*
IL-5	1. 14 (1.07,1.93)	2.04 (1.57,2.72)	1.65 (1.14,2.47)	0.0005*	0.1580	0.0818
IL-6	2. 77 (1.60,3.75)	28.10 (4.30,419.47)	4.34 (2.99,6.23)	<0.0001*	0.0114*	0.0020*
IL-8	16.35 (8.79,23.45)	117.87 (87.07,245.23)	114.43 (87.75,144.60)	<0.0001*	<0.0001*	0.2620
IL- 10	1.12 (1.07,1.35)	1.63 (1.39,2.25)	1.45 (1.29,1.63)	0.0001*	0.0021*	0.0338*
IL- 17	2.88 (1.82,3.90)	3.16 (2.50,4.44)	2.51 (2.28,3.54)	0.1049	0.8592	0.0515
IFN-γ	2.42 (2.08,2.68)	3.91 (2.42,7.93)	2.55 (1.14,4.23)	0.0159*	0.5994	0.0445*
TNF-α	1.82 (1.42,2.36)	2.91 (2.44,21.38)	6.70 (2.18,29.06)	<0.0001*	<0.0001*	0.9553

Data were not normally distributed; cytokine group comparisons were made using the Mann-Whitney U test, and results are presented as median (interquartile range); * denotes significance.

### 3.3 Diagnostic performance of selected cytokines

**Figure 1:**
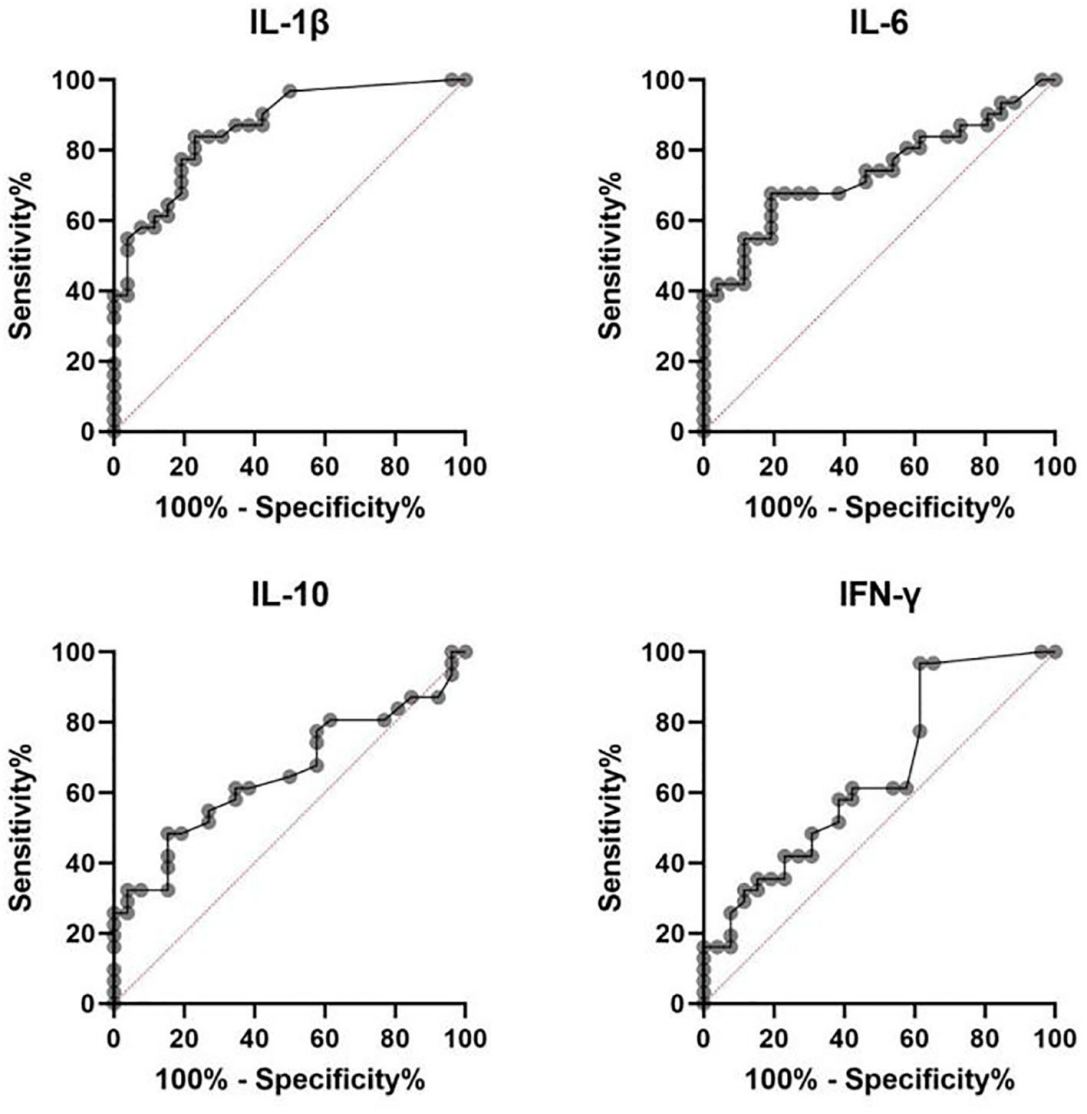
ROC curves of the four cytokines between PSS and POAG patients.

Receiver operating characteristic (ROC) curve analysis was conducted to assess the potential discriminatory ability of IL-1β, IL-6, IL-10, and IFN-γ in differentiating PSS from POAG. The results are presented in **Table 3** and **Figure 1**.

**Table 3:** ROC analysis results for aqueous humor cytokines between PSS and POAG patients.

Cytokines	AUC	95% CI	*P* value	Youden’s index	Cutoff	Sensitivity	Specificity
IL- 1β	0.867	0.750 - 0.942	<0.001	0.608	3.14	83.87	76.92
IL-6	0.739	0.606 - 0.846	0.0003	0.485	7.46	67.74	80.77
IL- 10	0.654	0.516 - 0.775	0.0359	0.330	1.64	48.39	84.62
IFN-γ	0.655	0.517 - 0.776	0.0036	0.496	1.34	96.77	38.46

Among the cytokines analyzed, IL-1β demonstrated the highest diagnostic performance, with an area under the curve (AUC) of 0.867 (95% CI: 0.750–0.942), sensitivity of 83.87%, and specificity of 76.92% at a cutoff of 3.14 pg/mL. IL-6 also showed moderate discriminative value (AUC = 0.739), followed by IFN-γ and IL-10.

### 3.4 Cytokine profiles stratified by clinical subgroups

Stratified analyses based on sex, age, presence of coexisting cataract, and CMV DNA status revealed no significant differences in aqueous humor cytokine levels among PSS subgroups (**Table 4**, all *P* > 0.05).

**Table 4:** Comparison of cytokine levels in PSS patients stratified by gender, age, coexisting cataract, and CMV-DNA detection.

Aqueous humor cytokines of PSS	Gender (males *vs* females) (*P* value)	Age (<50 *vs* ≥50) (*P* value)	Cataract (with *vs* without) (*P* value)	CMV-DNA (+ *vs* -) (*P* value)
IL- 1β	0.187	0.920	0.088	0.736
IL-5	0.670	0.857	0.632	0.921
IL-6	0.078	0.378	0.447	0.843
IL-8	0.935	0.337	0.708	0.620
IL- 10	0.584	0.214	0.417	0.487
IL- 17	0.273	0.631	0.662	0.170
TNF- α	0.919	0.496	0.130	0.858
IFN-γ	0.463	0.334	0.490	0.247

Cytokine group comparisons were made using the Mann-Whitney U test.

Among the 31 PSS patients, 17 (54.8%) tested positive for CMV DNA in aqueous humor by PCR. However, no statistically significant differences were observed in cytokine concentrations between CMV-positive and CMV-negative individuals (**Table 4**).

### 3.5 Correlation between cytokines and ophthalmic parameters

Correlation analysis between cytokine levels and ophthalmic measurements in the PSS group revealed that IL-6 was significantly and positively correlated with IOP (r = 0.395, *P* = 0.007) and relative endothelial cell loss (RECL; r = 0.453, *P* = 0.039) (**Table 5**). No other cytokines showed statistically significant correlations with CECD, visual field indices, or axial length. In multivariable regression analyses adjusted for age, sex, and intraocular pressure, IL-6 remained significantly associated with intraocular pressure and relative endothelial cell loss.

**Table 5:** Spearman correlation coefficients between cytokines and clinical parameters in PSS patients.

Ocular parameters result Mean ± SD/Median (Interquartile Range)		IL- 1β	IL-5	IL-6	IL-8	IL- 10	IL- 17	TNF-α	IFN-γ
Visual Acuity(LogMAR) 0.52(0.30~1.00)	*r* value	0.071	0.361	0.074	0.047	-0.080	0.254	-0.039	0.263
	*P* value	0.704	0.083	0.692	0.804	0. 669	0.168	0.836	0.160
IOP(mmHg) 32.9 ± 9.9	*r* value	-0.139	0.198	**0.395**	0.294	0.193	-0.251	0.134	-0.003
	*P* value	0.455	0.287	0.007*	0.109	0.298	0.173	0.473	0.989
Cup-to-Disc Ratio 0.8(0.6~0.9)	*r* value	-0.213	-0. 112	-0.082	-0.148	-0.180	0.085	0.238	-0.196
	*P* value	0.354	0.629	0.723	0.521	0.436	0.715	0.298	0.396
Axial length (mm) 24.33(23.68~25.20)	*r* value	-0.147	-0.128	0.014	0.117	0.024	-0.035	0.055	0.050
	*P* value	0.464	0.525	0.945	0.560	0.905	0.862	0.785	0.803
MD(dB) 13.78 ± 7.43	*r* value	-0.052	0.012	0.310	0. 141	-0.101	-0.027	-0.029	0.100
	*P* value	0.799	0.951	0.116	0.482	0.616	0.893	0.886	0.620
MS(dB) 12.37 ± 6.92	*r* value	0. 121	0.131	-0.076	-0.060	0.047	-0.115	-0.149	-0.207
	*P* value	0.557	0.525	0.712	0.770	0.819	0.576	0.469	0.311
CECD(cell/mm2) 2118 ± 348	*r* value	-0.156	-0.057	-0.379	-0.353	-0.145	-0.239	0.034	-0.161
	*P* value	0.499	0.805	0.074	0.116	0.531	0.297	0.882	0.485
RECL(cell/mm2) 483 ± 351	*r* value	-0.043	-0.302	**0.453**	0.004	-0.280	0.194	-0.303	0.113
	*P* value	0.853	0.184	0.039*	0. 987	0.220	0.400	0.182	0.625

Spearman’s rank correlation coefficients (r) are shown, with corresponding P values in parentheses. *P<0.05

IOP, (Intraocular Pressure); CECD, (Corneal Endothelial Cell Density); RECL, (Relative Endothelial Cell Loss); MD, (Mean Deviation), MS, (Mean Sensitivity); “*” indicates significant difference (r<0.05) Bold values indicate significant differences or correlations.

### 3.6 Inter-cytokine correlation analysis

Spearman’s rank correlation analysis revealed a complex cytokine regulatory network within the aqueous humor of patients with Posner-Schlossman syndrome (PSS) (**Table 6**). IL-6 exhibited strong positive correlations with both IL-8 (r = 0.688, *P* < 0.0001) and IL-10 (r = 0.487, *P* = 0.006), suggesting coordinated upregulation among these inflammatory mediators. Similarly, IL-8 was positively correlated with IL-10 (r = 0.506, *P* = 0.004) and IFN-γ (r = 0.404, *P* = 0.024), showing a significant positive correlation, which is consistent with (or may reflect) coordinated expression within a shared inflammatory pathway. IL-1β showed a positive correlation with IL-17 (r = 0.457, *P* = 0.010), but was negatively correlated with TNF-α (r = –0.491, *P* = 0.005), IL-1β levels showed a significant positive correlation with IL-17 and a significant negative correlation with TNF-α. This pattern of correlations suggests a complex, non-linear regulatory network among these cytokines in PSS, rather than a simple linear association. Additionally, IL-5 was negatively correlated with IFN-γ (r = –0.440, *P* = 0.013), reflecting a potential counterbalance between Th2- and Th1-type immune responses. Collectively, these findings highlight the intricate and dynamic cytokine interactions that may underlie the immunopathogenesis of PSS.

**Table 6:** Correlation analysis of different cytokines in aqueous humor.

Aqueous humor cytokines		IL- 1β	IL-5	IL-6	IL-8	IL- 10	IL- 17	TNF-α	IFN-γ
IL- 1β	*r* value	–	-0.122	0.125	-0.039	0.023	**0.457**	**-0.491**	-0.092
	*P* value	–	0.514	0.504	0.835	0. 904	**0.010**	**0.005**	0.621
IL-5	*r* value	-0.122	–	0.083	0.009	0.340	-0.229	0.190	**-0.440**
	*P* value	0.514	–	0.657	0.961	0.061	0.215	0.305	**0.013**
IL-6	*r* value	0.125	0.083	–	0.688	**0.487**	-0.029	-0.041	0.273
	*P* value	0.504	0.657	–	<0.0001*	**0.006**	0.876	0.825	0.138
IL-8	*r* value	-0.039	0.009	**0.688**	–	**0.506**	-0.171	0.097	**0.404**
	*P* value	0.835	0.961	<0.0001*	–	**0.004**	0.359	0.603	**0.024**
IL-10	*r* value	0.023	0.340	**0.487**	**0.506**	–	0.034	0.166	0.121
	*P* value	0. 904	0.061	**0.006**	**0.004**	–	0.855	0.373	0.517
IL-17	*r* value	**0.457**	-0.229	-0.029	-0.171	0.034	–	-0.208	0.027
	*P* value	**0.010**	0.215	0.876	0.359	0.855	–	0.262	0.887
TNF-α	*r* value	**-0.491**	0.190	-0.041	0.097	0.166	-0.208	–	0.015
	*P* value	**0.005**	0.305	0.825	0.603	0.373	0.262	–	0.934
IFN-γ	*r* value	-0.092	**-0.440**	0.273	**0.404**	0.121	0.027	0.015	–
	*P* value	0.621	**0.013**	0.138	**0.024**	0.517	0.887	0.934	–

Spearman’s rank correlation coefficients (*r*) are shown, with corresponding *P* values in parentheses. *P < 0.05. Bold values indicate significant differences or correlations.

## Discussion

This study delineates a unique inflammatory cytokine profile in the aqueous humor (AH) of patients with Posner-Schlossman syndrome (PSS), distinct from profiles in primary open-angle glaucoma (POAG) and cataract controls. Our findings underscore a central role for immune dysregulation in PSS pathogenesis.

Elevated levels of IL-1β, IL-6, IL-10, and IFN-γ in PSS AH were significant. Notably, in ROC analysis, these cytokines effectively discriminated PSS from POAG, highlighting their potential as diagnostic biomarkers in clinically ambiguous cases.

Approximately 55% of AH samples were CMV PCR-positive, yet cytokine levels did not differ significantly between PCR-positive and PCR-negative groups, aligning with prior findings (6). A negative PCR result does not exclude latent CMV infection, and the precise role of CMV—as an inflammatory activator or a bystander—in PSS remains to be defined. Therefore, there was no significant difference in cytokines between the CMV-positive group and the CMV-negative group.

The significant elevation of IL-1β, coupled with its positive correlation with IL-17 in PSS AH, is consistent with the involvement of Th17-mediated pathways—an underexplored mechanism in PSS. Given that IL-1β is a critical cytokine for the differentiation and functional regulation of human Th17 cells (12) and points to a pathway for chronic anterior chamber inflammation that extends beyond the classical Th1/Th2 imbalance.

IL-6 was notably elevated and demonstrated positive correlations with both intraocular pressure (IOP) and relative endothelial cell loss (RECL). This dual association supports the interpretation of IL-6 as a potential link between inflammatory activity and tissue damage, a concept corroborated by studies in related ocular conditions (8, 13). The co-elevation of IL-6, IL-8, and IL-10 reflects a coordinated, complex immune response within the anterior chamber.

IL-10 is secreted by TH2 cells (14). The rise in the anti-inflammatory cytokine IL-10 is consistent with a compensatory mechanism to modulate ongoing inflammation, potentially in response to viral triggers such as CMV (15, 16).Similarly, elevated IFN-γ levels, along with a negative correlation with IL-5, are indicative of a dominant Th1 response that could suppress Th2 activity in PSS, aligning with patterns observed in other infectious uveitides (17–19).

While TNF-α was elevated in both PSS and POAG, affirming its general association with glaucomatous processes (20–22), its negative correlation with IL-1β specifically in PSS implies a unique regulatory interaction within the PSS cytokine network that warrants further study.

This study is limited by a modest sample size, a consequence of PSS’s low incidence and recurrent nature, compounded by stringent inclusion and technical criteria. The lack of formal multiple-comparison correction represents a limitation of this exploratory study and warrants validation in larger, independent cohorts. Future multicenter studies employing more sensitive, high-throughput techniques are needed to validate these cytokine profiles and systematically explore their roles as biomarkers in PSS. It should be noted that despite statistically significant differences, considerable overlap in cytokine levels exists between PSS and POAG patients. Therefore, single-cytokine thresholds are unlikely to function as standalone diagnostic tools in clinical practice. Instead, these findings highlight cytokines that may contribute to differential disease profiling and could be incorporated into future multi-parameter or integrative diagnostic models.

## Conclusion

Our study found significantly increased levels of inflammatory factors such as IL-1β, IL-6, IL-10, IFN-γ in the aqueous humor of PSS patients, indicating that these molecules undergo significant changes in the aqueous humor of PSS and may be closely related to ocular lesions. The cytokines IL-6 in the aqueous humor exhibited a significant positive correlation with PSS key ophthalmic parameters, suggesting a potential relationship between ocular lesions and immune responses and inflammation in PSS patients. In general, there seems to be a stronger cytokine response in PSS than POAG, and the inflammation was promoted by a strong TH1 response in PSS. Further investigation into the role of cytokines and the balanced regulation of TH1/TH2/TH17 in the pathogenesis of PSS could provide novel perspectives and potential avenues for disease treatment and intervention.

## Data Availability

The original contributions presented in the study are included in the article/supplementary material. Further inquiries can be directed to the corresponding authors.
